# Can minimal clinically important differences in patient reported outcome measures be predicted by machine learning in patients with total knee or hip arthroplasty? A systematic review

**DOI:** 10.1186/s12911-022-01751-7

**Published:** 2022-01-20

**Authors:** Benedikt Langenberger, Andreas Thoma, Verena Vogt

**Affiliations:** Department of Health Care Management, Technische Universität Berlin, Berlin, Germany

## Abstract

**Objectives:**

To systematically review studies using machine learning (ML) algorithms to predict whether patients undergoing total knee or total hip arthroplasty achieve an improvement as high or higher than the minimal clinically important differences (MCID) in patient reported outcome measures (PROMs) (classification problem).

**Methods:**

Studies were eligible to be included in the review if they collected PROMs both pre- and postintervention, reported the method of MCID calculation and applied ML. ML was defined as a family of models which automatically learn from data when selecting features, identifying nonlinear relations or interactions. Predictive performance must have been assessed using common metrics. Studies were searched on MEDLINE, PubMed Central, Web of Science Core Collection, Google Scholar and Cochrane Library. Study selection and risk of bias assessment (ROB) was conducted by two independent researchers.

**Results:**

517 studies were eligible for title and abstract screening. After screening title and abstract, 18 studies qualified for full-text screening. Finally, six studies were included. The most commonly applied ML algorithms were random forest and gradient boosting. Overall, eleven different ML algorithms have been applied in all papers. All studies reported at least fair predictive performance, with two reporting excellent performance. Sample size varied widely across studies, with 587 to 34,110 individuals observed. PROMs also varied widely across studies, with sixteen applied to TKA and six applied to THA. There was no single PROM utilized commonly in all studies. All studies calculated MCIDs for PROMs based on anchor-based or distribution-based methods or referred to literature which did so. Five studies reported variable importance for their models. Two studies were at high risk of bias.

**Discussion:**

No ML model was identified to perform best at the problem stated, nor can any PROM said to be best predictable. Reporting standards must be improved to reduce risk of bias and improve comparability to other studies.

**Supplementary Information:**

The online version contains supplementary material available at 10.1186/s12911-022-01751-7.

## Introduction

Total hip arthroplasty (THA) and total knee arthroplasty (TKA), also referred to as total hip or total knee replacement [1], both subsumed under the term total joint arthroplasty (TJA) [2] reflect a common medical treatment in developed OECD countries. The rates for joint replacement are increasing rapidly in OECD countries within the last decades. From 2000 to 2013, a 35 percent increase in THAs and a roughly 100 percent increase in TKAs has been reported. However, there exist huge differences in TJA rates across countries within the OECD. Even though age is a strong predictor for the need of TJA, variations in population age are not the key driver of differences in TJA across countries [3]. For OECD countries, the trend in THAs is predicted to grow from 184 implants per 100,000 inhabitants (2015) up to 275 implants per 100,000 inhabitants in 2050 [4]. For TKA, the annual growth rate across OECD countries is estimated to be 5.5 percent per year [5]. Additionally, various international studies predict (highly) increasing rates of TKA and/or THA for countries such as the UK [6], Germany [7, 8], New Zealand [9], Sweden [10, 11], the US [12–14], Australia [15] or Taiwan [16]. However, it has been reported that up to 30 percent of patients undergoing TJA remain unsatisfied [17]. Thus, the question arises whether it is possible to select only patients for surgery that will also be satisfied afterwards.

Over the past decades, various standardized, patient reported measures have been developed to capture patient’s perception of their health or quality of life. These measures are referred to as patient reported outcome measures (PROMs). So far, the implementation of PROMs in clinical practice is rare [18]. There exist both indication/disease/condition specific PROMs such as for TKA [19] or THA [20], but also PROMs that measure generic health status [21]. Once measured before and after a medical treatment has been conducted, PROMs provide the possibility to evaluate whether a clinical treatment has led to an improvement *relevant* to the patient. Such improvements, specifically the smallest still relevant to the patient, are also referred to as minimal clinically important differences, short MCIDs [22, 23]. MCIDs for PROMs can be derived using different methods such as distribution-based methods [24, 25], anchor-based methods, or by expert consensus [23, 26].

Knowing that a substantial share of patients fail to achieve MCIDs after TJA [2, 27–29], it would be a huge advantage to know which patients will or will not achieve a MCID from a given treatment *before* the treatment is conducted [2]. If possible, accurate predictions can reduce costs for the healthcare system by preventing patients that will not benefit from treatment from unnecessarily receiving it, decreasing their risk associated with surgery and facilitate optimal resource allocation within the healthcare system.

An approach that could be exploited for predicting whether patients will receive MCIDs after TJA is machine learning (ML), a branch of artificial intelligence (AI) [30]. So far, ML methods have shown to be able to outperform more traditional methods such as regression techniques in various prediction tasks [31–34]. When ML is applied to prediction tasks, typically supervised ML is used [35]. Supervised ML is trained to perform predictions based on various variables (features), using training data where the outcome variables value is known (labelled) [35, 36]. Once trained, supervised ML is used to predict outcomes in unlabeled test data, which contains the same features as the training data. While predicting continuous outcomes in ML is referred to as regression, categorial outcome prediction is said to be classification [35]. However, there are various models available for application in a classification task. Among those, it is not clear a priori which model will perform best on a given task [35, 37]. Instead, researchers rely on trial and error, testing various models regarding their predictive performance, then selecting the model that performs best [35].

In contrast to traditional statistical prediction models such as logistic regressions (LRs), machine learning typically requires less human input, is less theory led and handles nonlinear relationships of variables [37], variable selection or interactions itself [27]. In application, traditional models are rather designed to identify associations than performing predictions [2]. However, there exist various distinctions of ML and traditional models, ranging from classifying anything else than traditional regression as ML [38] to defining the difference between statistical models and machine learning as a continuum, where a model is closer to ML the less human input (e.g. defining interactions, non-linear specifications) it requires [39]. Breiman [40] described ML and traditional models rather as two cultures. One culture aims to predict outcomes with given inputs without the aim to explain the relationship between inputs and outputs in detail. The other culture rather aims to model the relations between input and output correctly but is not deeply interested in achieving best predictive performance. For the reason of this study, we define ML as methods other than traditional LR or linear regression [38, 41], thus included models handle at least non-linear relationships, feature selection or interactions themselves. Consequentially, we distinguish from Christodoulou et al. [42] and also include LASSO (least absolute shrinkage and selection operator) models as ML into our review, setting the cut-off between ML and traditional models closer to the traditional models edge. However, some studies [27, 28] included have also performed LRs. LR results will not be presented in the results section of this paper.

ML techniques have already been applied and systematically reviewed in various prediction tasks in healthcare such as for sepsis prediction [43], psychiatric disorders prediction [41], neurosurgery outcomes [34], therapeutic outcomes in depression [44], and more. However, no systematic review summarizing the results for ML in the prediction of MCIDs in PROMs for patients undergoing TJA has been conducted so far.

Following the PICOTS scheme [45], our aim was to systematically review studies applying machine learning (I/C) in order to predict, based on pre-surgery data (T) from TKA/THA patients (P/S), whether or not patients that underwent TKA or THA (P) achieve a difference in pre- and post-surgery (T) PROM scores as high or higher as a derived MCID (O) (binary outcome, classification task).

## Methods

### Protocol and registration

The study was registered in the PROSPERO registry (ID: CRD42021229935) for systematic reviews on 4th of February 2021. No protocol has been published. We followed the Preferred Reporting Items for Systematic reviews and Meta-Analysis of Diagnostic Test Accuracy Studies (PRISMA-DTA) checklist for the structure of this systematic review.

### Eligibility criteria

Studies included in this review must satisfy the following criteria: patients included in the study underwent either total knee or total hip arthroplasty (of any etiology), or both; reported at least one PROM both before and at least one time after treatment; written in English or German; MCIDs were derived either anchor-based, distribution-based or through expert consensus (or referenced literature must include either of the three calculation categories); MCID calculation method must have been reported (at least in the referenced literature); predictions for MCIDs after treatment were performed; prediction models were based on machine learning; predictive performance was assessed using either area under the receiver operating curve (AUC)/c-statistic, J-statistic (Youden-Index), G-mean, F1-measure, sensitivity and specificity, or accuracy.

Studies were excluded from this review if they met the following criteria: case studies or reports; books; reviews; congress articles or presentations; only applied traditional statistical models; outcomes were not patient reported.

### Information sources

The search was conducted on November 2nd, 2021. The databases MEDLINE, PubMED Central (PMC), Web of Science Core Collection, Google Scholar and the Cochrane Library were searched. The initial search term consisted of several variations of four blocks that build up the search term components (see Additional file 1: Detailed search terms for each database searched). Each block consisted of several variations including MeSH terms, truncations, synonyms, acronyms, or related terms of the component aimed to be identified by each block. The first block consisted of terms related to supervised machine learning, which is the category of method necessary to be applied in the included papers. The second block consisted of terms indicating the use of PROMs, while the third consisted of terms related to MCIDs. Fourth and finally, terms related to total knee or hip arthroplasty were included into the search term.

### Search

Search terms are fully available in Additional file 1: Appendix 1.

### Study selection

**Fig. 1:**
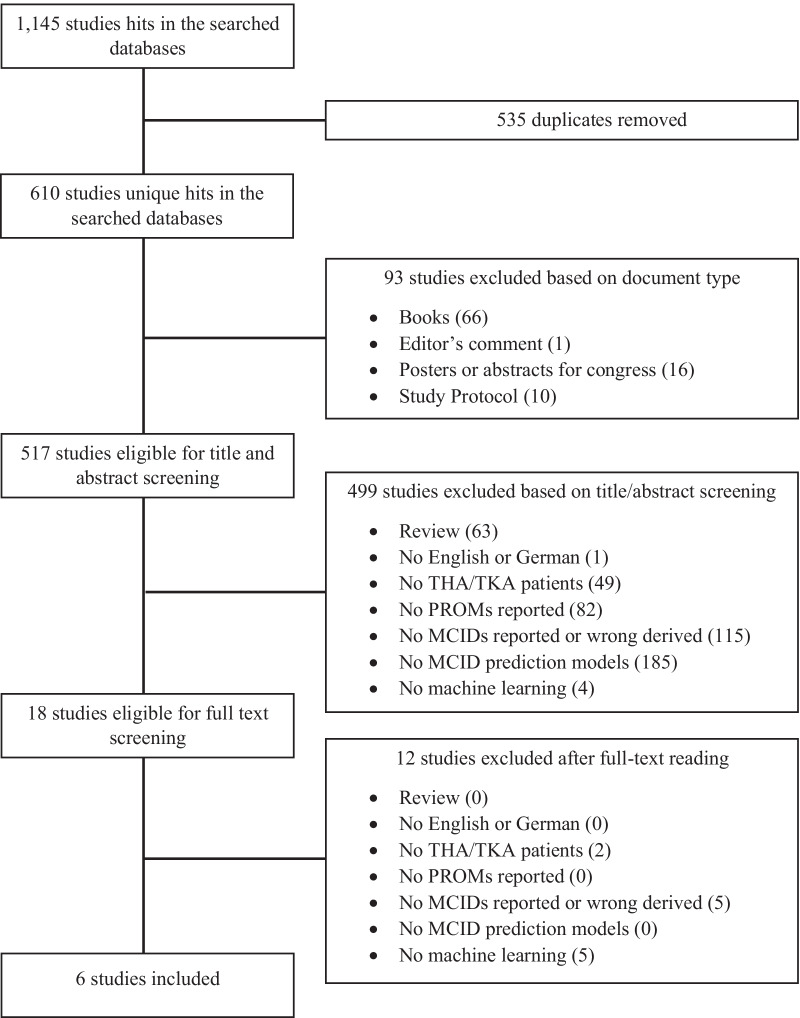
PRISMA (preferred reporting items for systematic reviews and meta-analysis) flowchart

After conducting the initial search, all papers identified on all databases were transferred to Citavi 6.7, a literature management software from the Swiss Academic Software GmbH, Wädenswil, Swiss. Next, all duplicates were removed. Further, all articles of excluded document type (see eligibility criteria) were removed. The remaining studies were screened on titles and abstracts. After excluding off-topic papers or papers in the wrong language (see Fig. 1), the rest of studies were read full text. After excluding studies that turned out to be subject to exclusion criteria / did not meet the inclusion criteria, the final papers included into the study were identified. The whole process was conducted by two researchers (BL, AT) independently. Differences in included papers in the different stages (identification, screening, eligibility, and inclusion) were discussed and settled by arguing in line with the inclusion and exclusion criteria. If differences could not be settled, a third researcher (VV) was available for consultation for final settlement. For each stage, we applied a low-threshold strategy. That is, if at least one of the search-conducting reviewers thought it is somehow possible that a paper has hit the inclusion criteria, even if one might assume objectively that it is unlikely, the paper was included for the next stage [46].

### Data collection and items collected

Once identified to be included into this systematic review, specific data from studies was collected standardized. That is, a previously constructed, standardized table including features relevant to interpret the predictive performance of the applied models was filled with the data reported by the respective study (Table 1). Specifically, the table includes the following items: Country of data origin, PROMs/MCID values, MCID calculation method, time-difference surgery to post-surgery PROM collection (months), number of observations, number of features, applied machine learning methods, ratio of training to test dataset, cross-validation applied in the training dataset, whether outlier detection and analysis were performed, whether missing value management was reported, whether feature preprocessing was performed, whether imbalanced data adjustment was performed, the AUC/c-statistic, J-statistic, F1-measure, sensitivity, specificity and accuracy, the Brier score, the best predictive model and best predicted PROM, and the predictive task (classification or regression).

**Table 1:** Extracted data of the included studies

Study	Fontana et al. [2]	Harris et al. [27]*	Huber et al. [28]*	Katakam et al. [59]	Zhang et al. [60]	Kunze et al. [29]
Country of data origin	US	US	UK	US	Not reported	US
Surgical procedure	THA/TKA	TKA	THA/TKA	TKA	TKA	THA
PROMs/MCID values	HOOS JR: 17.7 KOOS JR: 13.6 SF-36 (MCS + PCS): 5.0 (both)	KOOS Total: 91.8 KOOS JR: 20.8 KOOS Pain: 25.0 KOOS Symptoms: 14.3 KOOS ADL: 24.6 KOOS Quality of Life: 12.5 KOOS Recreation: 17.5	EQ VAS Hip: 11 EQ VAS Knee: 10 OHS: 8^a^ OKS: 7^a^	KOOS: MCID value not reported PROMIS Global PF: MCID value not reported PROMIS Global MH: MCID value not reported NRS Pain: MCID value not reported	SF-36 PCS: 10.0 SF-36 MCS: 5.0 WOMAC: 15.0	EQ VAS: Not reported
MCID calculation method	Anchor-based Distribution-based	Anchor-based	Distribution-based (VAS) Anchor-based (OKS, OHS)	Distribution-based	Anchor-based	Distribution-based^b^
Time-difference surgery to post-surgery PROM collection (months)	24	12	12	12	24	24
Number of observations	7,239 (THA) 6,480 (TKA)	587	30,524 (THA) 34,110 (TKA)	744	2840	616
Number of features^c^	66–97	6–106	81 (candidate predictors)	24 (candidate predictors)	18 (WOMAC); 19 (other PROMs)	8
Applied machine learning methods	LASSO Random forest Support vector machine	LASSO Gradient boosting machine Quadratic discriminant analysis	Extreme gradient boosting machine Random forest Multistep adaptive elastic net Neural network Naive Bayes k-nearest neighbours Boosted logistic regression	Stochastic gradient boosting Random forest Support vector machine Neural network Elastic-net penalized logistic regression	Support vector machine LASSO Random forest Extreme gradient boosting	Stochastic gradient boosting Random forest Support vector machine Neural network Elastic net penalized logistic regression
Ratio of training to test dataset	80:20	No test dataset	About 1:1 (dataset of the next year)	80:20	80:20	80:20
Cross-validation applied in the training dataset	Yes	Yes	Yes	Yes	Yes	Yes
Outlier detection and analysis performed?	Not reported	Not reported	Not reported	Not reported	Not reported	Not reported
Missing value management reported?	Yes	Not reported	Yes	Yes	Yes	Yes
Feature preprocessing performed?	Yes	Not reported	Not reported	Not reported	Not reported	Not reported
Imbalanced data adjustment performed?	Not reported	Not reported	Yes	Not reported	Yes	Not reported
AUC/c-statistic^e^	0.89 (not reported)	0.72 (not reported)^d^	Not reported on test data	0.77 (0.74–0.79)	SVM: 0.95 (0.94–0.97) XGB: 0.95 (0.94–0.97)	0.97 (0.94–0.99)
J-statistic	–	–	0.59 (not reported)	–	–	–
F1-measure	–	–	0.78 (not reported)	–	SVM: 0.85 (not reported) XGB: 0.86 (not reported)	–
Sensitivity	–	–	0.82 (not reported)	–	SVM: 93.1 (not reported) XGB: 95.6 (not reported)	–
Specificity	–	–	0.77 (not reported)	–	SVM: 86.8 (not reported) XGB: 84.9 (not reported)	–
Accuracy		–	0.79 (not reported), balanced accuracy	–	–	–
Brier Score^e^	Not reported	LASSO (KOOS Pain): 0.16 (not reported) LASSO (KOOS Symptoms): 0.17 (not reported) LASSO (KOOS ADL): 0.17 (not reported) GBM (KOOS PAIN): 0.16 (not reported) QDA (KOOS PAIN): 0.16 (not reported)	Not reported	0.15 (0.12–0.19)	SVM: 0.12 (not reported) XGB: 0.11 (not reported)	0.054 (0.047–0.062)
Best predictive model	Logistic LASSO Random forest	LASSO, Gradient boosting machine, QDA (Pain) LASSO (KOOS Symptoms + KOOS ADL)	Extreme gradient boosting	Neural Network Elastic-net penalized logistic regression	Support vector machine (SVM) Extreme gradient boosting (XGB)	Random forest
Best predictive PROM	SF-36 MCS	KOOS Pain KOOS Symptoms KOOS ADL	EQ VAS (hip)	KOOS	SF-36 MCS	EQ VAS
Predictive task	Classification	Classification	Classification	Classification	Classification	Classification

HOOS JR, Hip disability and osteoarthritis outcome score joint replacement; KOOS JR, Knee injury and osteoarthritis outcome score joint replacement; SF-36 MCS, Short form-36 mental component score; SF-36 PCS, Short form-36 physical component score; EQ, EuroQol; VAS, Visual analog scale; OKS, Oxford Knee Score; OHS, Oxford Hip Score; LASSO, Least absolute shrinkage and selection operator; AUC, area under the receiver operating curve; QDA, Quadratic discriminant analysis; ADL, Activities of daily life; JR, Joint replacement

*Also applied LR

^a^Value was taken from literature

^b^This value was calculated on postoperative score distribution

^c^Finally included in the models when not otherwise stated

^d^Result from the training dataset with fivefold cross validation as no AUC was reported on test data

^e^Confidence intervals (95% if not otherwise specified) in parenthesis

### Risk of bias assessment

Risk of bias assessment was conducted using PROBAST (Prediction model Risk of Bias ASsessment Tool). PROBAST includes a four-step approach. While steps one and two consider the review question and classification of the prediction model, step three assesses the risk of bias (ROB) and applicability, and finally step four closes with an overall judgement [47]. The assessment tool assesses participants, predictors, outcomes and analysis ROB as well as participants, predictors and applicability to the review question [45]. For PROBAST assessment, risk of bias is thus assessed both at study and outcome level.

### Summary measures

Outcomes of studies needed to be reported as AUC/c-statistics, J-statistics, G-mean, F1-measure, sensitivity and specificity, or accuracy.

### Synthesis of results

Due to the variety of models, features and included PROMs from which outcomes (MCIDs) were derived in the studies, no meta-analysis could be conducted.

## Results

### Study selection

Searches of the search terms were conducted on the previously mentioned databases (see section “Data sources”) on November the 2nd, 2021. Searches in PubMed Central, MEDLINE, Web of Science Core Collection, Google Scholar and Cochrane Library yielded 139, 355, 314, 237 and 100 hits, respectively, resulting in 1145 hits in total. All hits were transferred to Citavi 6.7. 610 unique titles were identified. 93 were excluded based on document type (see eligibility criteria). After screening title and abstract of the remaining 517 studies, 18 studies underwent full text screening. Thereof, six titles were included after full-text reading (see Fig. 1).

From the 18 articles screened for full text, two studies were excluded due to including the wrong study population from the perspective of this review. One study [48] included knee arthroscopies and one [49] patients with osteochondral allograft for cartilage defects. Next, five studies [50–54] did not calculate MCIDs, even though they researched PROMs in TJA patients. Finally, six studies [26, 55–58] aimed to make comparable predictions as searched for this review except failing to apply machine learning.

### Study characteristics

#### Machine learning models applied

The studies included applied various machine learning techniques, while two of them [2, 27] also included LRs. One for comparison with ML [27], the other defined LR as ML [28]. The number of included ML techniques varied from three [2] to seven [28]. Overall, the four most commonly applied algorithms across studies were random forest [2, 28, 29, 59, 60], gradient boosting machine (GBM)^1^ [21, 28, 29, 59, 60], support vector machine (SVM) [2, 29, 59, 60] and LASSO [2, 27, 60]. We reported, for each study, the model that performed best on the test dataset on the studies main outcome metric, which was the AUC for all studies except for Huber et al. [28], where J-statistic was the favored outcome on the test dataset. In case only a validation dataset was used and no test dataset [27], the main outcome on the validation dataset was reported. For the model(s) performing best on the main outcome, we also reported all other outcomes reported in the studies.

#### Sample size, data origin and number of features

Sample size varied widely across studies. While Harris et al. [27] only included 587 TKAs (both for training and validation) in their analysis, Huber et al. [28] included 34,110 individuals with TKA for model development and 34,406 TKAs for testing. For THA, they used 30,524 and 31,905 individuals for training and testing, respectively. The other studies ranged in between (see Table 1). While Huber et al. [28] used data from NHS treated patients among patients treated in multiple centers (all run by NHS), Katakam et al. [59] used data from five sites, Harris et al. [27] included three sites (VA medical centers). Zhang et al. [60], Kunze et al. [29] and Fontana et al. [2] only exploited data from one site.

Due to the different datasets utilized, the number of features initially available to the models varied across studies. Fontana et al. [2] had access to 51 initial variables in their “before surgery” setting. However, as 25 of them were categorial, the number of features (including the categories dummies) included in their analysis must have been higher but was not reported. Further, the number of features used by each model was not reported. Harris et al. [27] came up with 106 variables out of which models were able to select the variables relevant for predictions. Their final models included six to 106 variables, depending on algorithm and PROM. Huber et al. [28] had access to 81 variables out of which models were able to select their individual number of predictors, however the final number of features per model was not reported. Kunze et al. [29] initially had access to eleven variables to be included in predictive models. After recursive feature selection, they came up with eight variables included into the final models. Katakam et al. [59] initially had access to 24 variables (including dummies for categorial variables). They did not report the final number of variables after feature selection exploiting the random forest. Finally, Zhang et al. [60] included 18 variables for the WOMAC and 19 variables for the other PROMs in their final prediction models.

#### Training, validation, and testing

All included studies used some approach to perform training and validation of their developed models. However, studies applied different approaches to do so. Harris et al. [27] performed cross-validation with bootstrapping on the training dataset to assess their models’ performance (internal validation) but did not exploit any unforeseen test dataset. Huber et al. [28] used cross-validation in the training dataset for model selection and tested their models on an independent test dataset of the subsequent year. The remaining studies split their dataset beforehand randomly into training and test data. While the proportions of training to dataset were 20:80 in Fontana et al. [2], Kunze et al. [29] and Zhang et al. [60], Katakam et al. [59] split the dataset into 70 percent training and 30 percent test data. During model training, all studies applied cross-validation to perform hyperparameter tuning and model selection. Then, performance evaluation was executed on the test dataset which was completely unforeseen by the algorithms. Huber et al. [28] and Zhang et al. [60] applied upsampling (a replication of the minority class to receive a balanced training dataset) in the training dataset to account for disproportions across outcome groups.

#### Predictive performance

To evaluate models’ predictive performance, papers reported different performance indicators. For performance comparison in this section, only performance reported on the test dataset is included since it is indicative of model generalization, while performance assessment on training data could be biased due to overfitting. That is, models fit the training data disproportionally well but have poor generalization on test data [35]. As an exception, in case of development studies, performance on training data was reported in case cross-validation was applied to account for overfitting.

Most of the studies reported the AUC as main performance measure. The AUC is calculated as the area under the receiver operator curve, and the receiver operator curve is a plot of sensitivity against false positive rate (1-specificity) of a given predictive tool using different decision thresholds for categorizing outcomes as either positive or negative [61]. AUC/c-statistic values are classified as fail (0.5–0.59), poor (0.6–0.69), fair (0.7–0.79), good (0.8–0.89) or excellent (0.9–1.0) [27, 62]. The best model in Harris et al. [27] and Katakam et al. [59] performed fair, the best model in Fontana et al. [2] good and the models of Kunze et al. [29] and Zhang et al. [60] excellent on the test/validation sample (see Table 1). Unfortunately, Huber et al. [28] did not report AUC results for the test sample. Applying their models to the test sample, they came up with a J-statistics of 0.59. The J-statistics (Youden Index) is the sum of sensitivity and specificity minus one [63]. If the proportion of predicted positives in the true positives group is higher than in the true negatives group, the value is always above zero [63].

#### Utilized PROMs and MCID derivation

PROMs used varied widely across studies. Across all included studies, sixteen different PROMs have been applied to KA and six to HA patients, respectively. No PROM was utilized in all studies. All included studies except Kunze et al. [29] included at least three PROMs. Kunze et al. [29] only included a generic PROM. Harris et al. [27] only included treatment-specific PROMs, the remaining studies included both generic and treatment specific PROMs.

Fontana et al. [2] included four PROMs in their paper, namely SF-36 physical component score (PCS), SF-36 mental component score (MCS), Hip Disability and Osteoarthritis Outcome Score for Joint replacement (HOOS JR) and the Knee Disability and Osteoarthritis Outcome Score for Joint replacement (KOOS JR). While the former are generic health status scores with either an additional focus on physical (PCS) or on mental health (MCS) [64], the latter are knee or hip specific scores [65]. Harris et al. [27] included KOOS Total, JR and the subscales KOOS pain, symptoms, activities of daily living (ADL), quality of life (QoL) and recreation. Huber et al. [28] included the EQ-5D-3L and EQ VAS (both general health) as well as the Oxford Knee Score (OKS) and Oxford Hip Score (OHS), which are both disease specific, whereas Kunze et al. [29] only included the generic EQ VAS. Katakam et al. [59] included the KOOS as disease-specific PROM and the Patient Reported Outcomes Measurement Information System (PROMIS) Global PF, PROMIS Global MH [66] and numerical rating scale for pain (NRS Pain). Finally, Zhang et al. [60] reported the Western Ontario and McMaster Universities Osteoarthritis Index (WOMAC) as well as SF-36 PS and SF-36 MCS.

MCIDs can be calculated either anchor-based, distribution-based or based on evidence from previous studies [67]. All studies used distribution-based or anchor-based methods or referred to such. While for HOOS JR and KOOS JR they adopted values based on anchor-based methods from the literature, Fontana et al. [2] determined the score for SF-36 PCS and MCS themselves based on distribution-based methods. Harris et al. [27] calculated MCIDs applying anchor-based methods, with the Self-Administered Patient Satisfaction Scale (SAPS) as anchor. To determine the score for each PROM which discriminates patient satisfaction best, they used the Youden index. Huber et al. [28] calculated MCIDs using distribution-based methods (half a standard deviation) for EQ VAS (preoperative score) and referred to the literature for OHS and OKS, respectively. Kunze et al. [29] also defined MCIDs based on distribution-based methods as half of a standard deviation. However, in contrast to the other studies, they used postoperative scores instead of preoperative scores to determine MCIDs. Katakam et al. [59] derived all MCIDs by distribution-based methods. Zhang et al. [60] referred to the literature which based the MCID calculation on anchor-based methods for MCID derivation of all PROMs.

#### Variable importance

Out of the six included studies, five [2, 28, 29, 59, 60] reported variable importance. If studies reported variable importance for different algorithms, for simplicity, only the variable importance for best performing algorithms as well as the best predictive PROM will be reported. Among those, preoperative PROM scores were the most predictive variables in all studies. In four studies [2, 28, 59, 60], a depression-indicating variable was among the top five most important predictors. Further, three studies [2, 28, 59] had at least two other PROMs or PROM subscales ranging among the top five predictors for MCIDs. As Kunze et al. [29] did not include more than one PROM in their study, it was not possible to have other PROM scores among the best predictor variables.

#### Missing data

Studies facilitated different strategies to deal with missing data. Fontana et al. [2] handled numeric missing variables by imputation to the mean, while for categorial variables, an extra class was created for missing values, exploiting information of missing values. Huber et al. [28] removed all patients with missing values and variables with variance close to or at zero. Kunze et al. [29] and Katakam et al. [59] performed multiple imputation for variables with less than 30 percent missing values. Kunze et al. [29] excluded one variable with more than 30 percent missing values. Zhang et al. [60] reported two variables with few missing values and applied imputation with mean values. Harris et al. [27] did not report missing values. A detailed overview of missing values for each study is given in Additional file 2: Missing data values for included studies.

### Risk of bias assessment

Risk of bias assessment within studies was conducted using PROBAST (Prediction model Risk of Bias ASsessment Tool), which assesses the bias for the prediction tools. PROBAST includes a four-step approach. While steps one and two consider the review question and classification of the prediction model, step three assesses the risk of bias (ROB) and applicability, and finally step four closes with an overall judgement [47]. Table 2 represents the suggested tabular presentation for PROBAST results by Wolff et al. [47]. A guidance on how to perform PROBAST is also given at the website probast.org, and in Moons et al. [45] (Additional file 3: Probast assessment of all included studies).

**Table 2:** PROBAST ROB and applicability assessment results* for all included studies following the suggested tabular presentation by Wolff et al. [47]

	ROB				Applicability			Overall	
Study	Participants	Predictors	Outcome	Analysis	Participants	Predictors	Outcome	ROB	Applicability
Fontana et al. [2]	+	+	+	+	+	+	+	+	+
Harris et al. [27]	+	+	+	−	+	+	+	−	+
Huber et al. [28]	+	+	+	−	+	+	+	−	+
Katakam et al. [59]	+	+	+	+	+	+	+	+	+
Zhang et al. [60]	+	+	+	+	+	+	+	+	+
Kunze et al. [29]	+	+	+	+	+	+	+	+	+

ROB, Risk of bias

* + indicates low ROB/low concern regarding applicability; − indicates high ROB/high concern regarding applicability; and ? indicates unclear ROB/unclear concern regarding applicability

After conducting the PROBAST assessment, four studies were free of ROB, while two were of high risk of ROB. All studies were applicable, that is, populations, predictors and outcomes fit this reviews purpose [47]. All studies had in common that predictors were not excluded from the outcome definition, yielding a possible bias introduction [45]. As the case in this setting, the outcome (MCID) is partially defined by pre-surgery PROMs, a predictor also applied in the prediction models. Therefore, bias was not assumed to be introduced by pre-operative PROMs due to the nature of the study setting. Next, all studies determined the outcome with knowledge of predictor information, i.e. predictors were known as the outcome was determined [45]. That is inherent to the study setting and is not assumed to introduce any bias. Further, almost all studies had events per variable (EVP) – that is, the number of participants with the least often reported outcome (MCID vs. no MCID) over the number of candidate predictors – below the recommended value for machine learning of 200 by Moons et al. [45]. However, Moons et al. [45] refer to van der Ploeg et al. [68] for the value of 200. van der Ploeg et al. [68] indicated a number of EVP ≥ 200 if studies AUC was reported on the training dataset and not tested on a validation dataset, calculating the bias (or optimism) as difference between the AUC on the training and on the validation dataset. As stated, all studies at least applied cross-validation to account for overfitting, thus no study reported outcome metrics only on the training dataset without any validation [68]. Consequentially, EVP was not considered to be able to introduce bias in all studies.

However, two studies [27, 28] were subject to risk of bias beyond that common traits. Harris et al. [27] did not repot how missing values were handled, nor if there were any, resulting in a high ROB judgement. Note that, following Moons et al. [45], Harris et al. [27] can only be rated as model development study but not as validation study, as they only performed internal validation (cross-validation), but no validation on an external test dataset [45].

Huber et al. [28] did not report calibration of models as outcome metric, a potential ROB in the analysis domain [45]. Additionally, important metrics such as AUC, which is commonly reported across all other studies included in this review, was only reported on the training dataset. However, on the test dataset, it was not reported, and it was further not stated why it was not reported on the test dataset. Furthermore, Huber et al. [28] dropped all participants with missing values, another potential for bias introduction [45]. Moreover, Huber et al. [28] did only imprecisely describe the study population. Instead of TKA/THA patients, which are the patients the NHS England PROMs dataset consists of [69], this study only reported their participants to be knee or hip replacement patients. The reader unfamiliar with the dataset may conclude that the dataset includes hip/knee replacement patients other than TKA/THA. Consequentially, Huber et al. [28] was characterized as being at high ROB.

## Discussion

This paper was the first to systematically review approaches predicting MCIDs in patient reported outcome measures for patients undergoing total hip or knee arthroplasty. As summarized, all six included papers were published within the last two years. Given that a substantial amount of patients undergoing joint replacement remain unsatisfied afterwards [17] and/or do not achieve an MCID in general reported health [28, 29] or condition-specific PROMs [27], there is a need of creating, evaluating, and implementing approaches to accurately identify patients which would remain unsatisfied after surgery. The actuality of papers published indicates that the problem is recently getting attention and that modern approaches like ML are being exploited to identify such patients.

All studies included various models, and models were partially common among studies (mainly random forest, GBM, LASSO, SVM, neural networks, elastic net LR). However, no type of model clearly outperformed others across studies. This is in line with Hastie et al. [35], who state that there is no ML algorithm that is known a priori to perform best on a given problem and thus only application will show which algorithm (with which tuning parameters) is superior in the specific setting. For example, random forest was applied in five studies, but only performed as best model in two. However, Kunze et al. [29] and Katakam et al. [59] described that they performed feature selection with a random forest. It could be that predictive performance was biased in these studies so that the random forest ended up with features best fitting for that method, but other models could have performed better with other features. Nevertheless, among all studies, performance of the applied ML models did not differ too much. Interestingly and counterintuitively, neither the number of features included, nor the sample size seemed to influence predictive performance to a large extend. Specifically, the best predictive model on AUC [29] was developed with the second smallest sample (n = 616) and with the fewest number of features available compared to all other studies. Overall, ML models’ performance was well in the respective prediction task, indicated by the fact that three out of five studies that reported AUC on test data reached good or excellent performance [27, 29, 60]. The AUC is an appropriate metric for measuring discrimination and to our knowledge the only metric applicable to imbalanced data without being biased [70] – a highly valuable characteristic in datasets with MCIDs as outcomes. Besides that, it has its disadvantages. Specifically, in practice, it is often necessary to have outcome metrics that reflect the performance of an algorithm at a specific sensitivity specificity trade-off [71]. In that context, other metrics are more appropriate and might be reported on a balanced test dataset in case of imbalanced data [70]. Four out of six studies [2, 27, 29, 59] did only report the AUC as discrimination metric, while one [28] did completely rely on other metrics on test data. This reflects potential for improvement. The AUC is an important performance indicator and therefore should be reported by all studies. Common metrics such as F1-measure, G-mean or J-statistic could be additionally reported without much extra effort, and we encourage authors to do so.

PROMs included differed widely across studies. Due to the heterogeneity of datasets and PROMs, conclusions about which PROM can be predicted best are not possible yet. However, EQ VAS [28, 29] and SF-36 MCS [2, 60] were each two times the best predictable PROMs in the six included studies. Interestingly, Fontana et al. [2] and Zhang et al. [60] found that MCIDs for SF-36 MCS, a mental health PROM, could be better predicted than somatic ones (HOOS JR, KOOS JR, WOMAC, SF-36 PCS) in TJA patients. In general, there is a certain advantage of using generic PROMs like EQ VAS or EQ-5Ds over indication-specific PROMs such as KOOS or HOOS. With generic (i.e. disease unspecific) PROMs, medical outcomes of procedures such as surgeries for TKA or THA can be made comparable with other healthcare interventions, facilitating cost-effectiveness analysis and improving decision makers information on allocating healthcare resources [72].

MCID calculations for PROMs were performed according to established methods. However, two studies [29, 59] did not report the MCIDs threshold value. This should be a standardized procedure. Reporting MCID values is relevant because then they can be compared  with MCID values from other studies. Recent evidence suggests that MCIDs for PROMs are highly variable and can differ substantially across study populations, calculation methods or even within the same calculation method (e.g. anchor-based calculation, but using different anchors might yield different results) [73]. It remains unclear whether the MCID calculation method has an impact on ML performance to predict MCIDs in total joint arthroplasty patients. This is a relevant question and should be subject to further research. That being said, three studies [2, 28, 29] defined at least some MCIDs based on distributions. Even though distribution-based methods are common practice, it is not clear that they detect differences that really matter to patients rather than being an arbitrary set threshold based on observed data. MCIDs calculated with anchor-based techniques may be preferred as they stronger focus on the patients perspective [74].

Additionally, studies should report all relevant outcome measures, including calibration. For one study [28], the lack of reporting calibration was one factor for categorizing it as high ROB. Further, even if not a source of bias in the ROB [45], confidence interval reporting is highly important as it helps to understand the certainty of the estimates. However, we found that only half of the studies reported confidence intervals at least for the main outcome, showing high potential for improvement. Furthermore, only two studies [28, 60] performed imbalanced data adjustment. This might be crucial when using other performance metrics than the AUC [70] or when models are sensitive to balanced datasets during training [28]. We therefore recommend to at least test balancing classes and report it, even if it yields no benefit. The same holds for feature preprocessing (e.g. variable transformations), a description of the country where the study took place (not reported in Zhang et al. [60]), a precise description of the study population (imprecise in Huber et al. [28]) and reporting how missing data was handled. No study reported how outliers were handled. We advise all machine learning algorithm developers to assess their studies using the IJMEDI checklist for machine learning applications in medicine as described in Cabitza et al. [75]. It helps to avoid such problems. All studies but one [27] properly applied model validation, feature selection and hyperparameter tuning on the training dataset and reported their final results on an unforeseen test dataset, even though one study [28] did not report all important metrics on the test dataset. However, the result that 5 of 6 studies applied their models to independent test datasets provides strong evidence that the predictive performance in forecasting whether patients will receive an MCID or not after TJA is reasonable to believe. Additionally, all papers discussed the clinical utility and limitations of their applications. Two [27, 28] compared their ML models with traditional approaches, Zhang et al. [60] discussed the lack of comparison with traditional models as a limitation.

Two studies [27, 28] had a high risk of bias. Main problems in the studies with high risk of bias could have been easily handled through transparent and reasonable reporting [35]. Further, given that ML prediction models performance is typically assessed using AUC [41, 43, 76], studies should apply this metric to increase transparency and comparability with other studies’ results. Especially when used as the *main* metric for assessing performance in the training dataset [28], it should not be omitted in the test dataset.

However, this study comes with limitations. First, as a result of the limited number of studies included for the purpose of this review, and due to the results of ROB both within and across studies, the performance of ML models in the stated context based on this body of evidence should be interpreted with caution. Second, we did not search the literature database Scopus due to a lack of access and might have potentially missed relevant studies. Fortunately, evidence suggests high precision of our used search engines in identifying relevant literature [77, 78]. Third, as model parameter tuning is up to the researcher [35], it can always lead to inferior performing models. We were not able to control hyperparameter tuning in the studies and therefore rely on the subjective performance of the individual researchers of the studies in doing so. This also holds that different methods might outperform others when tuned more sophisticated. Therefore, we also recommend reporting various specifications of the used models with the best tuned model indicated. Fourth and finally, we did not compare machine learning methods to other prediction methods. It might be that other prediction tools such as logistic regression or pre-surgery PROMs themselves might perform as good, better, or worse than machine learning. This needs further investigation. ML studies may therefore include other methods as baseline/comparison models.

## Conclusion

Given the promising results of models’ performance of the included studies, ML-based applications to support informed decision making as well as to implement an objective instance in shared decision making between clinicians and patients undergoing TKA or THA should be considered for practical implementation. Discussed issues of risk of bias and underreporting must be eliminated in future research to derive transparent and unbiased results. Especially, important metrics such as the AUC, calibration and uncertainty should be reported standardized across and consistently within studies for better comparability. Further, dataset preparation especially with respect to an unforeseen dataset is crucial when it comes to performance assessment and should be applied in every study. Relevant checklists are available to ensure high quality of studies applying ML in healthcare.

## Supplementary Information

**Additional file 1**. Appendix 1: Search Terms.

**Additional file 2**. Appendix 2: Missing data per variable for all included studies.

**Additional file 3**. Appendix 3: PROBAST assessment of all included studies.

## Data Availability

All relevant materials are available in the supporting files (e.g. PRISMA-DTA).

## Footnotes

1 Note that several GBM variations such as stochastic gradient boosting, extreme gradient boosting or gradient boosting are summarized under the umbrella of GBM.

## References

[1] Kahlenberg CA, Richardson SS, Gruskay JA, Cross MB (2020). Trends in utilization of total and unicompartmental knee arthroplasty in the United States. J Knee Surg.

[2] Fontana MA, Lyman S, Sarker GK, Padgett DE, MacLean CH (2019). Can machine learning algorithms predict which patients will achieve minimally clinically important differences from total joint arthroplasty?. Clin Orthop Relat Res.

[3] Organisation for Economic Co-operation and Development (2015). Health at a glance 2015: OECD indicators.

[4] Pabinger C, Lothaller H, Portner N, Geissler A (2018). Projections of hip arthroplasty in OECD countries up to 2050. Hip Int.

[5] Pabinger C, Lothaller H, Geissler A (2015). Utilization rates of knee-arthroplasty in OECD countries. Osteoarthritis Cartil.

[6] Culliford D, Maskell J, Judge A, Cooper C, Prieto-Alhambra D, Arden NK (2015). Future projections of total hip and knee arthroplasty in the UK: results from the UK Clinical Practice Research Datalink. Osteoarthritis Cartil.

[7] Pilz V, Hanstein T, Skripitz R (2018). Projections of primary hip arthroplasty in Germany until 2040. Acta Orthop.

[8] Rupp M, Lau E, Kurtz SM, Alt V (2020). Projections of primary TKA and THA in Germany from 2016 through 2040. Clin Orthop Relat Res.

[9] Hooper G, Lee AJJ, Rothwell A, Frampton C (2014). Current trends and projections in the utilisation rates of hip and knee replacement in New Zealand from 2001 to 2026. N Z Med J.

[10] Nemes S, Gordon M, Rogmark C, Rolfson O (2014). Projections of total hip replacement in Sweden from 2013 to 2030. Acta Orthop.

[11] Nemes S, Rolfson O, W-Dahl A, Garellick G, Sundberg M, Kärrholm J, Robertsson O (2015). Historical view and future demand for knee arthroplasty in Sweden. Acta Orthop.

[12] Patel A, Pavlou G, Mújica-Mota RE, Toms AD (2015). The epidemiology of revision total knee and hip arthroplasty in England and Wales: a comparative analysis with projections for the United States. A study using the National Joint Registry dataset. Bone Joint J.

[13] Sloan M, Premkumar A, Sheth NP (2018). Projected volume of primary total joint arthroplasty in the U.S. 2014 to 2030. J Bone Joint Surg Am.

[14] Singh JA, Yu S, Chen L, Cleveland JD (2019). Rates of total joint replacement in the United States: future projections to 2020–2040 using the national inpatient sample. J Rheumatol.

[15] Inacio MCS, Graves SE, Pratt NL, Roughead EE, Nemes S (2017). Increase in total joint arthroplasty projected from 2014 to 2046 in australia: a conservative local model with international implications. Clin Orthop Relat Res.

[16] Kumar A, Tsai W-C, Tan T-S, Kung P-T, Chiu L-T, Ku M-C (2015). Temporal trends in primary and revision total knee and hip replacement in Taiwan. J Chin Med Assoc.

[17] Gandhi R, Davey JR, Mahomed NN (2008). Predicting patient dissatisfaction following joint replacement surgery. J Rheumatol.

[18] Nelson EC, Eftimovska E, Lind C, Hager A, Wasson JH, Lindblad S (2015). Patient reported outcome measures in practice. BMJ (Clinical Research ed.).

[19] Ramkumar PN, Harris JD, Noble PC (2015). Patient-reported outcome measures after total knee arthroplasty: a systematic review. Bone Joint Res.

[20] Gagnier JJ, Huang H, Mullins M, Marinac-Dabić D, Ghambaryan A, Eloff B (2018). Measurement properties of patient-reported outcome measures used in patients undergoing total hip arthroplasty: a systematic review. JBJS Rev.

[21] Harris K, Dawson J, Gibbons E, Lim CR, Beard DJ, Fitzpatrick R, Price AJ (2016). Systematic review of measurement properties of patient-reported outcome measures used in patients undergoing hip and knee arthroplasty. Patient Relat Outcome Meas.

[22] Jaeschke R, Singer J, Guyatt GH (1989). Measurement of health status. Control Clin Trials.

[23] McGlothlin AE, Lewis RJ (2014). Minimal clinically important difference: defining what really matters to patients. JAMA.

[24] Escobar A, Quintana JM, Bilbao A, Aróstegui I, Lafuente I, Vidaurreta I (2007). Responsiveness and clinically important differences for the WOMAC and SF-36 after total knee replacement. Osteoarthritis Cartil.

[25] Norman GR, Sloan JA, Wyrwich KW (2003). Interpretation of changes in health-related quality of life: the remarkable universality of half a standard deviation. Med Care.

[26] Berliner JL, Brodke DJ, Chan V, SooHoo NF, Bozic KJ (2017). Can preoperative patient-reported outcome measures be used to predict meaningful improvement in function after TKA?. Clin Orthop Relat Res.

[27] Harris AHS, Kuo AC, Bowe TR, Manfredi L, Lalani NF, Giori NJ (2021). Can machine learning methods produce accurate and easy-to-use preoperative prediction models of one-year improvements in pain and functioning after knee arthroplasty?. J Arthroplasty.

[28] Huber M, Kurz C, Leidl R (2019). Predicting patient-reported outcomes following hip and knee replacement surgery using supervised machine learning. BMC Med Inform Decis Mak.

[29] Kunze KN, Karhade AV, Sadauskas AJ, Schwab JH, Levine BR (2020). Development of machine learning algorithms to predict clinically meaningful improvement for the patient-reported health state after total hip arthroplasty. J Arthroplasty.

[30] Russell SJ, Norvig P, Davis E, Edwards D. Artificial intelligence: a modern approach. Boston, Columbus, Indianapolis, New York, San Francisco, Upper Saddle River, Amsterdam, Cape Town, Dubai, London, Madrid, Milan, Munich, Paris, Montreal, Toronto, Delhi, Mexico City, Sao Paulo, Sydney, Hong Kong, Seoul, Singapore, Taipei, Tokyo: Pearson; 2016.

[31] Senders JT, Arnaout O, Karhade AV, Dasenbrock HH, Gormley WB, Broekman ML, Smith TR (2018). Natural and artificial intelligence in neurosurgery: a systematic review. Neurosurgery.

[32] Azimi P, Mohammadi HR, Benzel EC, Shahzadi S, Azhari S, Montazeri A (2015). Artificial neural networks in neurosurgery. J Neurol Neurosurg Psychiatry.

[33] Azimi P, Benzel EC, Shahzadi S, Azhari S, Mohammadi HR (2016). The prediction of successful surgery outcome in lumbar disc herniation based on artificial neural networks. J Neurosurg Sci.

[34] Senders JT, Staples PC, Karhade AV, Zaki MM, Gormley WB, Broekman MLD (2018). Machine learning and neurosurgical outcome prediction: a systematic review. World Neurosurg.

[35] Hastie T, Tibshirani R, Friedman J (2009). The elements of statistical learning.

[36] Jiang F, Jiang Y, Zhi H, Dong Y, Li H, Ma S (2017). Artificial intelligence in healthcare: past, present and future. Stroke Vasc Neurol.

[37] Boulesteix AL, Schmid M (2014). Machine learning versus statistical modeling. Biom J.

[38] He H, Garcia EA (2008). Learning from imbalanced data. IEEE Trans Knowl Data Eng.

[39] Beam AL, Kohane IS (2018). Big data and machine learning in health care. JAMA.

[40] Breiman L (2001). Statistical modeling: the two cultures. Stat Sci.

[41] Bracher-Smith M, Crawford K, Escott-Price V (2021). Machine learning for genetic prediction of psychiatric disorders: a systematic review. Mol Psychiatry.

[42] Christodoulou E, Ma J, Collins GS, Steyerberg EW, Verbakel JY, van Calster B (2019). A systematic review shows no performance benefit of machine learning over logistic regression for clinical prediction models. J Clin Epidemiol.

[43] Fleuren LM, Klausch TLT, Zwager CL, Schoonmade LJ, Guo T, Roggeveen LF (2020). Machine learning for the prediction of sepsis: a systematic review and meta-analysis of diagnostic test accuracy. Intensive Care Med.

[44] Lee Y, Ragguett R-M, Mansur RB, Boutilier JJ, Rosenblat JD, Trevizol A (2018). Applications of machine learning algorithms to predict therapeutic outcomes in depression: a meta-analysis and systematic review. J Affect Disord.

[45] Moons KGM, Wolff RF, Riley RD, Whiting PF, Westwood M, Collins GS (2019). PROBAST: a tool to assess risk of bias and applicability of prediction model studies: explanation and elaboration. Ann Intern Med.

[46] Valderas JM, Kotzeva A, Espallargues M, Guyatt G, Ferrans CE, Halyard MY (2008). The impact of measuring patient-reported outcomes in clinical practice: a systematic review of the literature. Qual Life Res.

[47] Wolff RF, Moons KGM, Riley RD, Whiting PF, Westwood M, Collins GS (2019). PROBAST: a tool to assess the risk of bias and applicability of prediction model studies. Ann Intern Med.

[48] Kunze KN, Polce EM, Rasio J, Nho SJ (2021). Machine learning algorithms predict clinically significant improvements in satisfaction after hip arthroscopy. Arthroscopy.

[49] Ramkumar PN, Karnuta JM, Haeberle HS, Owusu-Akyaw KA, Warner TS, Rodeo SA (2021). Association between preoperative mental health and clinically meaningful outcomes after osteochondral allograft for cartilage defects of the knee: a machine learning analysis. Am J Sports Med.

[50] Bloomfield RA, Broberg JS, Williams HA, Lanting BA, McIsaac KA, Teeter MG (2021). Machine learning and wearable sensors at preoperative assessments: functional recovery prediction to set realistic expectations for knee replacements. Med Eng Phys.

[51] Felix J, Becker C, Vogl M, Buschner P, Plötz W, Leidl R (2019). Patient characteristics and valuation changes impact quality of life and satisfaction in total knee arthroplasty—results from a German prospective cohort study. Health Qual Life Outcomes.

[52] Jayakumar P, Bozic KJ (2020). Advanced decision-making using patient-reported outcome measures in total joint replacement. J Orthop Res.

[53] Pua YH, Poon CLL, Seah FJT, Thumboo J, Clark RA, Tan MH (2019). Predicting individual knee range of motion, knee pain, and walking limitation outcomes following total knee arthroplasty. Acta Orthop.

[54] Twiggs J, Miles B, Roe J, Fritsch B, Liu D, Parker D (2021). Can TKA outcomes be predicted with computational simulation? Generation of a patient specific planning tool. Knee.

[55] Hart AJ, Sabah SA, Sampson B, Skinner JA, Powell JJ, Palla L (2014). Surveillance of patients with metal-on-metal hip resurfacing and total hip prostheses: a prospective cohort study to investigate the relationship between blood metal ion levels and implant failure. J Bone Joint Surg Am.

[56] Stiegel KR, Lash JG, Peace AJ, Coleman MM, Harrington MA, Cahill CW (2019). Early experience and results using patient-reported outcomes measurement information system scores in primary total hip and knee arthroplasty. J Arthroplasty.

[57] Weber M, Zeman F, Craiovan B, Thieme M, Kaiser M, Woerner M (2019). Predicting outcome after total hip arthroplasty: the role of preoperative patient-reported measures. Biomed Res Int.

[58] Yeo MGH, Goh GS, Chen JY, Lo N-N, Yeo S-J, Liow MHL (2020). Are Oxford hip score and western ontario and McMaster universities osteoarthritis index useful predictors of clinical meaningful improvement and satisfaction after total hip arthroplasty?. J Arthroplasty.

[59] Katakam A, Karhade AV, Collins A, Shin D, Bragdon C, Chen AF (2021). Development of machine learning algorithms to predict achievement of minimal clinically important difference for the KOOS-PS following total knee arthroplasty. J Orthop Res.

[60] Zhang S, Lau BPH, Ng YH, Wang X, Chua W (2021). Machine learning algorithms do not outperform preoperative thresholds in predicting clinically meaningful improvements after total knee arthroplasty. Knee Surg Sports Traumatol Arthrosc.

[61] Pepe MS (2000). Receiver operating characteristic methodology. J Am Stat Assoc.

[62] Hosmer DW, Lemeshow S (2010). Applied logistic regression.

[63] Youden WJ (1950). Index for rating diagnostic tests. Cancer.

[64] Jenkinson C, Stewart-Brown S, Petersen S, Paice C (1999). Assessment of the SF-36 version 2 in the United Kingdom. J Epidemiol Commun Health.

[65] Hung M, Saltzman CL, Greene T, Voss MW, Bounsanga J, Gu Y (2018). Evaluating instrument responsiveness in joint function: the HOOS JR, the KOOS JR, and the PROMIS PF CAT. J Orthop Res.

[66] Hays RD, Schalet BD, Spritzer KL, Cella D (2017). Two-item PROMIS® global physical and mental health scales. J Patient Rep Outcomes.

[67] Revicki D, Hays RD, Cella D, Sloan J (2008). Recommended methods for determining responsiveness and minimally important differences for patient-reported outcomes. J Clin Epidemiol.

[68] van der Ploeg T, Austin PC, Steyerberg EW (2014). Modern modelling techniques are data hungry: a simulation study for predicting dichotomous endpoints. BMC Med Res Methodol.

[69] NHS England. The national patient reported outcome measures (PROMS) programme 2018. 10.1177/0141076816677856 PMC5154408 27923896

[70] Jeni LA, Cohn JF, La Torre F (2013). Facing imbalanced data recommendations for the use of performance metrics. Int Conf Affect Comput Intell Interact Workshops.

[71] Halligan S, Altman DG, Mallett S (2015). Disadvantages of using the area under the receiver operating characteristic curve to assess imaging tests: a discussion and proposal for an alternative approach. Eur Radiol.

[72] Tew M, Dalziel K, Clarke P, Smith A, Choong PF, Dowsey M (2020). Patient-reported outcome measures (PROMs): can they be used to guide patient-centered care and optimize outcomes in total knee replacement?. Qual Life Res.

[73] Maredupaka S, Meshram P, Chatte M, Kim WH, Kim TK (2020). Minimal clinically important difference of commonly used patient-reported outcome measures in total knee arthroplasty: review of terminologies, methods and proposed values. Knee Surg Relat Res.

[74] Clement ND, Bardgett M, Weir D, Holland J, Gerrand C, Deehan DJ (2018). What is the minimum clinically important difference for the WOMAC index after TKA?. Clin Orthop Relat Res.

[75] Cabitza F, Campagner A (2021). The need to separate the wheat from the chaff in medical informatics: introducing a comprehensive checklist for the (self)-assessment of medical AI studies. Int J Med Inform.

[76] Shung D, Simonov M, Gentry M, Au B, Laine L (2019). Machine learning to predict outcomes in patients with acute gastrointestinal bleeding: a systematic review. Dig Dis Sci.

[77] Bramer WM, Giustini D, Kramer B, Anderson P (2013). The comparative recall of Google Scholar versus PubMed in identical searches for biomedical systematic reviews: a review of searches used in systematic reviews. Syst Rev.

[78] Gehanno J-F, Rollin L, Darmoni S (2013). Is the coverage of Google Scholar enough to be used alone for systematic reviews. BMC Med Inform Decis Mak.

