# Supplementary material for: Can minimal clinically important differences in patient reported outcome measures be predicted by machine learning in patients with total knee or hip arthroplasty? A systematic review
Source: BMC Med Inform Decis Mak. 2022 Jan 20;22:18. doi: 10.1186/s12911-022-01751-7 (PMC8772225; doi:10.1186/s12911-022-01751-7)
Supplement: Supplementary file 2 — Additional file 2. Appendix 2: Missing data per variable for all included studies. [file 12911_2022_1751_MOESM2_ESM.pdf]

Appendix 2: Missing data per variable for all included studies.

A) Fontana et al 2019.

Feature	Missing (%)
Baseline SF-36 physical component score (PCS)	5%
Baseline SF-36 mental component score (MCS)	5%
Baseline SF-36 physical functioning score	1%
Baseline SF-36 role physical score	2%
Baseline SF-36 bodily pain score	1%
Baseline SF-36 general health score	2%
Baseline SF-36 vitality score	1%
Baseline SF-36 social functioning score	1%
Baseline SF-36 role emotional score	3%
Baseline SF-36 mental health score	1%
Baseline HOOS JR or KOOS JR score	1%
Baseline HOOS or KOOS pain score	1%
Baseline HOOS or KOOS symptom score	2%
Baseline HOOS or KOOS ADL score	1%
Baseline HOOS or KOOS sports/rec score	15%
Baseline HOOS or KOOS QOL score	3%
Baseline WOMAC pain score	2%
Baseline WOMAC stiffness score	1%
Baseline WOMAC function score	3%
Baseline LEAS	2%
Baseline pain VAS	3%
Baseline fatigue VAS	2%
Baseline general health VAS	3%
Baseline EQ-5D score	3%
Baseline expectations survey score	25%
Number of procedures before index surgery date during inpatient stay	0.05%
ASA score	0.04%
Day of week of baseline survey	0%
Month of year of baseline survey	0%
Surgeon	0.05%

Doctor	0.01%
Day of week of index surgery	0%
Month of year of index surgery	0%
Attended presurgery class (or not)	32%
CCS procedure codes before surgery during inpatient stay	0.05%

Own table, adopted from Fontana et al 2019.

B) Katakam et al 2020.

Variable	Missing (%)
Hemoglobin	21.3%
White blood cell count	21.3%
Platelet count	21.3%
Creatinine level	22.4%
Median household income	1.5%
High school education	0.9%
Unemployment rate	0.9%
Preoperative PROMIS Global PF	6.3%
Preoperative PROMIS Global MH	6.3%
Preoperative VAS Pain	6.3%
1-year postoperative KOOS	28.9%

Own table.

C) Harris et al 2020.

No missing values reported.

D) Huber et al 2019.

Variables with missing values not reported.

E) Kunze et al 2020.

Variable	Missing (n)
MCID	28
Preoperative opioids	2
Smoking history	2
Diabetes	2
Comorbidities	2

Preoperative health state	26
Peoperative ROM*	197

Own table. *Only variable with > 30% missing values and was therefore excluded from the Analysis (Kunze et al 2020).

F) Zhang et al 2021.

Variable	Missing (n)
BMI	9
Preoperative pain score	5

Own table.
